# Critical Appraisal and Data Extraction for Systematic Reviews of Prediction Modelling Studies: The CHARMS Checklist

**DOI:** 10.1371/journal.pmed.1001744

**Published:** 2014-10-14

**Authors:** Karel G. M. Moons, Joris A. H. de Groot, Walter Bouwmeester, Yvonne Vergouwe, Susan Mallett, Douglas G. Altman, Johannes B. Reitsma, Gary S. Collins

**Affiliations:** 1 Julius Center for Health Sciences and Primary Care, UMC Utrecht, Utrecht, The Netherlands; 2 Department of Primary Care Health Sciences, New Radcliffe House, University of Oxford, Oxford, United Kingdom; 3 Centre for Statistics in Medicine, University of Oxford, Botnar Research Centre, Windmill Road, Oxford, United Kingdom

## Abstract

Carl Moons and colleagues provide a checklist and background explanation for critically appraising and extracting data from systematic reviews of prognostic and diagnostic prediction modelling studies.

*Please see later in the article for the Editors' Summary*

**Summary Points**

- Publications on clinical prediction models have become abundant for both prognostic and diagnostic purposes. Systematic reviews of these studies are increasingly required to identify and critically appraise existing evidence.
- No specific guidance exists to help frame a well-defined review question and determine which details to extract and critically appraise from primary prediction modelling studies.
- Existing reporting guidelines, quality assessment tools, and key methodological publications were examined to identify seven items important for framing the review question and 11 domains to extract and critically appraise the primary included studies.
- Together these items and domains form the **CH**ecklist for critical **A**ppraisal and data extraction for systematic **R**eviews of prediction **M**odelling **S**tudies (CHARMS).

## Introduction

Prediction models, both diagnostic and prognostic, are becoming increasingly abundant in the medical literature [1]–[3]. Diagnostic models are aimed at calculating the probability that an individual has a certain disorder, such as deep vein thrombosis [4],[5], ankle fractures [6], or conjunctivitis [7]. Prognostic prediction models concern the prediction of the probability or risk of the future occurrence of a particular outcome or event in individuals at risk of such an event. Prognostic models may involve models for individuals with a particular health condition, such as prediction of recurrence or death after diagnosis of breast cancer [8] or mortality after cardiac surgery [9], but also includes models for predicting the occurrence of future outcomes in apparently healthy individuals such as the risk of developing a coronary event [10] or type 2 diabetes mellitus [11].

There are over 100 models for predicting outcome after brain trauma [12], over 60 models for breast cancer prognosis [13], 45 models for cardiovascular events after being diagnosed with diabetes [14], 43 models for predicting prevalent and incident type 2 diabetes [15], and 20 models for predicting prolonged intensive care stay after cardiac surgery [16]. Furthermore, prediction models are increasingly being appraised and recommended for formal risk assessment in clinical guidelines [17],[18].

To evaluate the proliferation of prediction models, systematic reviews are necessary and led to the formation of the Cochrane Collaboration Prognosis Reviews Methods Group [19],[20]. Since then, search strategies for identifying prognostic and diagnostic prediction model studies have been developed [21]–[23], validated, and further refined [24].

However, no published checklists support the design of systematic reviews of prediction modeling studies, or what to extract and how to appraise primary prediction modelling studies. Existing guidance for synthesizing studies of prognostic factors [25],[26] does not address studies of multivariable prediction models. Instead, reviews of prediction model studies have created their own checklist [2],[12],[14],[15],[27]–[30], with variable inclusion of key details.

Our aim was to design a **CH**ecklist for critical **A**ppraisal and data extraction for systematic **R**eviews of prediction **M**odelling **S**tudies (CHARMS). The checklist is designed to help form a review question for and appraisal of all types of primary prediction modelling studies, including, regressions, neural network, genetic programming, and vector machine learning models [1]–[3],[12],[14],[15],[27]–[30]. Some items, such as “selection of predictors during multivariable modelling” and “model presentation”, are somewhat more specific to regression approaches. The checklist is not intended for systematic reviews of primary studies of prognostic factors, for which we refer to the QUIPS tool [25],[26], nor is it intended for prediction model impact studies in which, in principle, a comparative (intervention) design is used [1],[31],[32]. Box 1 shows the types of prediction modelling studies for which the CHARMS checklist was developed.

**Box 1. Types of Prediction Modelling Studies**

- *Prediction model development studies without external validation* aim to develop a prognostic or diagnostic prediction model from the dataset at hand: the development set. Such studies commonly aim to identify important predictors for the outcome under study, assign mutually adjusted weights per predictor in a multivariable analysis, develop a final prediction model, and quantify the predictive performance (e.g., discrimination, calibration, classification) of that model in the development set. As model overfitting may occur, particularly in small datasets, development studies ideally include internal validation using some form of data re-sampling techniques, such as bootstrapping, jack-knife, or cross-validation, to quantify any optimism in the predictive performance of the developed model.
- *Prediction model development studies with external validation in independent data* have the same aim as the previous type, but the development of the model is followed by quantifying the model's predictive performance in participant data external to the development dataset. This may be done in participant data collected by the same investigators, commonly using the same predictor and outcome definitions and measurements, but from a later time period (temporal or narrow validation), or by other investigators in another hospital or country (geographical or broad validation).
- *External model validation studies with or without model updating* aim to assess and compare the predictive performance of an existing prediction model using new participant data that were not used to develop the prediction model and possibly adjust or update the model in case of poor performance based on the validation data.

Prediction studies exploring which predictors independently contribute to the prediction of a particular prognostic or diagnostic outcome as well as studies aimed at quantifying the impact of using a prediction model (on, e.g., clinical decision making, patient outcomes, or cost-effectiveness of care) relative to not using the model may also be considered in a systematic review of prognostic and diagnostic prediction models [2]. However, data extraction and critical appraisal of those types of prediction studies is very different as they have different aims, designs, and reporting issues compared to studies developing or validating prediction models. Therefore, here we explicitly focus on reviews of studies aimed at developing, validating, or updating a prediction model.

## Development of the Checklist

We developed our checklist based on published risk of bias tools, existing critical appraisal checklists for systematic reviews of randomised therapeutic trials and diagnostic test accuracy research, methodological recommendations for conduct and reporting of prediction model research, and data extraction sheets used in published reviews of prediction modelling studies after contacting authors.

First, we reviewed the existing reporting guidelines for other types of clinical research including CONSORT, REMARK, STARD, STROBE, GRIPS [33]–[37] and for the reporting of systematic reviews (PRISMA) [38]. Furthermore, we considered existing quality assessment tools including the Cochrane Risk of Bias tool [39] for randomised therapeutic studies, QUADAS (and QUADAS-2) for diagnostic accuracy studies [40],[41], and the QUIPS checklist for appraisal of prognostic factor studies [25],[26]. We then reviewed published systematic reviews of prediction models and prognostic factor studies, along with the checklists or quality appraisal criteria used in those reviews [12],[27]–[29],[42]–[46]. Finally, we identified key methodological literature discussing recommended approaches for the design, conduct, analysis, and reporting of prediction models, followed by a search of the corresponding reference lists [3],[19],[31],[32],[37],[47]–[59].

Initial pilot versions of this checklist were presented and discussed at the annual Cochrane Prognosis Methods Group meetings and workshops from 2010–2014, held during the Cochrane Collaboration Colloquia, and modified based on feedback received during these meetings. Consecutive iterations of the checklist were applied, tested, and modified in various systematic reviews of prediction models [2],[14]–[16],[29],[60]–[62], which ultimately led to the current checklist. For the actual reporting of systematic reviews of prediction models, we refer to the PRISMA statement [38].

## The Checklist

The checklist contains two parts. Table 1 summarises key items to guide the framing of the review aim, search strategy, and study inclusion and exclusion criteria. Table 2 and Text S1 describe the overall domains and specific items within each domain to extract from the reports of primary prediction modelling studies in light of the review question, with a view to evaluate risk of bias and applicability.

**Table 1:** Key items to guide the framing of the review aim, search strategy, and study inclusion and exclusion criteria.

Item	Comments and examples
**1. Prognostic versus diagnostic prediction model**	Define whether the aim is to review models to predict:
	• Future events: prognostic prediction models
	• Current (disease) status: diagnostic prediction models
**2. Intended scope of the review**	Define intended scope of the review and intended purpose of the models reviewed in it. Examples:
	• Models to inform physicians' therapeutic decision making
	• Models to inform referral to or withholding from invasive diagnostic testing
**3. Type of prediction modelling studies (see also ** **Box 1** **)**	Define the type of prediction modelling studies to include. Examples of study types (Box 1):
	• Prediction model development without external validation in independent data
	• Prediction model development with external validation in independent data
	• External model validation, possibly with model updating
**4. Target population to whom the prediction model applies**	Define the target population relevant to the review scope. Examples:
	• Women with diagnosed breast cancer
	• Healthy adult men in the general population
**5. Outcome to be predicted**	Define the outcome of interest to be predicted:
	• Specific future event, such as a fatal or non-fatal coronary heart disease
	• Specific diagnostic target disease, such as presence of lung embolism
**6. Time span of prediction**	Define over what specific time period the outcome is predicted (prognostic models only). Example:
	• Event within a specific time interval, such as event within 3 months, 1 year, or 10 years
**7. Intended moment of using the model**	The systematic review may focus on models to be used at a specific moment in time. Examples:
	• Models to be used at the moment of diagnosis of a particular disease
	• Models to be used preoperatively to predict the risk of postoperative complications
	• Models to be used in asymptomatic adults to detect undiagnosed type 2 diabetes mellitus

**Table 2:** Relevant items to extract from individual studies in a systematic review of prediction models for purposes of description or assessment of risk of bias or applicability.

Domain	Key items	General	Applicability	Risk of bias
**Source of data**	• Source of data (e.g., cohort, case-control, randomised trial participants, or registry data)		X	X
**Participants**	• Participant eligibility and recruitment method (e.g., consecutive participants, location, number of centres, setting, inclusion and exclusion criteria)	X	X	
	• Participant description	X	X	
	• Details of treatments received, if relevant		X	X
	• Study dates	X	X	
**Outcome(s) to be predicted**	• Definition and method for measurement of outcome		X	X
	• Was the same outcome definition (and method for measurement) used in all patients?			X
	• Type of outcome (e.g., single or combined endpoints)	X	X	
	• Was the outcome assessed without knowledge of the candidate predictors (i.e., blinded)?			X
	• Were candidate predictors part of the outcome (e.g., in panel or consensus diagnosis)?			X
	• Time of outcome occurrence or summary of duration of follow-up		X	
**Candidate predictors (or index tests)**	• Number and type of predictors (e.g., demographics, patient history, physical examination, additional testing, disease characteristics)	X		
	• Definition and method for measurement of candidate predictors		X	X
	• Timing of predictor measurement (e.g., at patient presentation, at diagnosis, at treatment initiation)		X	
	• Were predictors assessed blinded for outcome, and for each other (if relevant)?			X
	• Handling of predictors in the modelling (e.g., continuous, linear, non-linear transformations or categorised)			X
**Sample size**	• Number of participants and number of outcomes/events	X		
	• Number of outcomes/events in relation to the number of candidate predictors (Events Per Variable)			X
**Missing data**	• Number of participants with any missing value (include predictors and outcomes)	X		X
	• Number of participants with missing data for each predictor			X
	• Handling of missing data (e.g., complete-case analysis, imputation, or other methods)			X
**Model development**	• Modelling method (e.g., logistic, survival, neural networks, or machine learning techniques)	X		
	• Modelling assumptions satisfied			X
	• Method for selection of predictors for inclusion in multivariable modelling (e.g., all candidate predictors, pre-selection based on unadjusted association with the outcome)			X
	• Method for selection of predictors during multivariable modelling (e.g., full model approach, backward or forward selection) and criteria used (e.g., p-value, Akaike Information Criterion)			X
	• Shrinkage of predictor weights or regression coefficients (e.g., no shrinkage, uniform shrinkage, penalized estimation)		X	X
**Model performance**	• Calibration (calibration plot, calibration slope, Hosmer-Lemeshow test) and Discrimination (C-statistic, D-statistic, log-rank) measures with confidence intervals		X	
	• Classification measures (e.g., sensitivity, specificity, predictive values, net reclassification improvement) and whether a priori cut points were used			X
**Model evaluation**	• Method used for testing model performance: development dataset only (random split of data, resampling methods, e.g., bootstrap or cross-validation, none) or separate external validation (e.g., temporal, geographical, different setting, different investigators)			X
	• In case of poor validation, whether model was adjusted or updated (e.g., intercept recalibrated, predictor effects adjusted, or new predictors added)		X	X
**Results**	• Final and other multivariable models (e.g., basic, extended, simplified) presented, including predictor weights or regression coefficients, intercept, baseline survival, model performance measures (with standard errors or confidence intervals)	X	X	
	• Any alternative presentation of the final prediction models, e.g., sum score, nomogram, score chart, predictions for specific risk subgroups with performance	X	X	
	• Comparison of the distribution of predictors (including missing data) for development and validation datasets			X
**Interpretation and Discussion**	• Interpretation of presented models (confirmatory, i.e., model useful for practice versus exploratory, i.e., more research needed)	X	X	
	• Comparison with other studies, discussion of generalizability, strengths and limitations	X	X	

*Risk of bias* refers to the extent that flaws in the design, conduct, and analysis of the primary prediction modelling study lead to biased, often overly optimistic, estimates of predictive performance measures such as model calibration, discrimination, or (re)classification (usually due to overfitted models). *Applicability* refers to the extent to which the primary study matches the review question, and thus is applicable for the intended use of the reviewed prediction model(s) in the target population.

### Guidance to frame the review question, search strategy, and study inclusion and exclusion criteria

Table 1 addresses seven key issues (i.e., prognostic versus diagnostic prediction model, intended scope of the review, type of prediction modelling studies [see also Box 1], target population to whom the prediction model applies, outcome to be predicted, time span of the prediction, and intended moment of using the model) that are helpful for systematic reviewers to frame the review question and design the review. A focused review question enables researchers to develop a tailored search strategy and to define the inclusion and exclusion criteria—and thus the applicability—of primary studies included in the review.

At the outset, the reviewer should decide whether the aim is to review prognostic or diagnostic models (item 1) and define the scope of the review (item 2). It is then important to decide whether to include model development studies, model validation studies, or both (item 3 and Box 1). For example, if the review aims to assess the performance of a specific prediction model, then only external validation studies of that model are applicable for the review.

Defining the target population of the prediction model(s) under review (item 4) and the outcome(s) to be predicted (item 5) are related items that are particularly important to indicate the potential usefulness and application of the review results. For example, relevance to physicians and patients is enhanced by models that predict patient-relevant outcomes, such as death, pain, or recurrence of disease, rather than those that predict process outcomes such as duration of hospital stay or intermediate outcomes, except when there is a clear and established causal association with a subsequent patient-relevant outcome (e.g., predicting CD4 count instead of complications in patients with HIV [47]).

Prognostic models commonly have a better predictive accuracy for short-term outcomes than for long-term outcomes (item 6) [63]. However, predicting long-term outcomes may sometimes be more relevant from a patient perspective, though this is obviously questionable in very elderly individuals [64].

Finally, clarifying when the model is intended to be used is important to define what sorts of models are relevant for the review (item 7). Models that incorporate predictors collected after this predefined time point are inappropriate. For example, if the aim is to review prognostic models to preoperatively predict the risk of developing post-operative pain within 48 hours after hip surgery, studies including intraoperative characteristics are not useful.

In Box 2 we give various examples of potential review questions of both prognostic and diagnostic models.

**Box 2. Examples of Systematic Reviews of Prognostic or Diagnostic Prediction Models with Different Aims**

**Reviews of prediction models for specific target populations (development and/or validation)**

- Existing models for predicting the risk of having undiagnosed or developing (incident) type 2 diabetes in adults [15].
- Prognostic models for activities of daily living, to be used in the early post-stroke phase [46].

**Reviews of prediction models for specific outcomes in a target population (development and/or validation)**

- Prognostic models for survival, for independence in activities of daily living, and for getting home, in patients with acute stroke [27].
- Prediction models for diagnosis of venous thromboembolism in patients suspected of having the disease [28].

**Review of the performance of one or more specific models (validation)**

- Predictive performance of the EuroSCORE for operative mortality following cardiac surgery when validated in other patient samples [139].
- Relative predictive performance of specific prognostic models for occurrence of cardiovascular disease when applied in general populations [44].

**Reviews of all existing models in a particular clinical field (development and/or validation)**

- Existing prognostic models in reproductive medicine [29].
- Existing prognostic models in the traumatic brain setting [12].

**Review of methods and reporting of prediction models (development and/or validation)**

- Quality of reporting of diagnostic and prognostic modelling studies published in high impact general medical journals or in a specific time period [2],[48].
- Reporting and methods used to develop prognostic models in cancer [60].

**Review of added value of specific predictor or updating of a specific model (development and/or validation)**

- Added predictive value of C-reactive protein to the Framingham risk scores [134].
- Added predictive value of carotid imaging markers to existing cardiovascular predictors in the general population [140].

### Relevant items to extract from individual studies

The key items to be extracted from each primary study are grouped within 11 domains. Similar to critical appraisal checklists for systematic reviews of randomised therapeutic and diagnostic accuracy studies, these address potential sources of bias in the primary studies and issues that may affect the applicability of the results in relation to the intended use of the prediction models.

#### Source of data

Data from cohort, nested case-control, or case-cohort studies are recommended for prognostic model development and validation studies, and cross-sectional designs for diagnostic modelling studies [47],[58],[59],[65]–[67]. Clearly, a prospective cohort design is preferable, as it enables optimal measurement of predictors and outcome. However, prospective studies evaluating (validating) the performance of an existing model predicting a long-term outcome, e.g., ten-year survival, may be too costly or the results insufficiently timely. Retrospective cohorts typically have a longer follow-up period, but usually at the expense of poorer data quality and unmeasured predictors [13]. A non-nested case-control design, as opposed to a nested case-control or case-cohort design, is inappropriate for developing a prediction model since the design does not enable calculation of absolute risks and thus yields incorrect estimates of model intercept or baseline hazard [65]–[68].

Randomised trials are a specific form of a prospective cohort study and thus share its advantages. However, restrictive eligibility criteria for entry into the trial may hamper generalizability of the prediction model. Furthermore, treatments shown to be effective in the trial should be acknowledged and possibly accounted for in the prediction model, as they may affect the predictive accuracy of the prognostic model [47],[56]. Finally, data from existing registries (e.g., administrative or routine care hospital databases) are increasingly used in prediction modelling studies. However, such databases are especially prone to missing data and missing important predictors, which can affect the predictive accuracy and applicability of the resulting prediction model [31],[56],[58],[69].

#### Participants

The participant recruitment method is important to establish whether the study population is representative of the target population. A review of 83 diagnostic prediction models for detection of ovarian malignancy found that studies often sampled participants non-consecutively [70], increasing the risk of bias due to selective sampling [42],[56],[71]. Also, it is important to ascertain from the publication whether all included participants were eventually used to develop or validate the prediction model [15],[56]. Selective inclusion based on data availability is likely to influence the predictive accuracy of the prediction model, as study data are seldom missing completely at random but are often missing in a selective and biased way (see section below on missing data).

Participant description, including inclusion and exclusion criteria, study setting (e.g., primary or secondary care), and number of centres, is important to allow for proper assessment of the applicability and thus generalizability of the study findings [40],[41],[56],[72]. For reviews of a single model that has been validated in different study samples, differences or heterogeneity in study design, sample characteristics, and setting that will affect the performance of the prediction model should be determined. For example, prediction models developed in secondary care perform less well when evaluated in a primary care setting [73],[74]. Reviews of prognostic models for patients with breast cancer [56] and patients with lower back pain [75] have identified that participant characteristics were often poorly reported.

The performance of prediction models may also vary depending on whether the study participants have received any treatment (including self-administered interventions) that may modify the outcome occurrence. It is therefore important to determine whether the review addresses treated or non-treated individuals or both, and whether the treatment effects (i.e., treatment predictors) were handled appropriately in the models. Finally, the dates of participant recruitment provide important information on the technological state of the tests and treatments used, and the lifestyle factors at that time. The predictive accuracy of models may change over time and require periodic updating [76], as was done for the QRISK models [77].

#### Outcome to be predicted

The definition and measurement of the outcome event (prognostic models) or the target disease (diagnostic models) in the primary studies should correspond to the outcome definition of the systematic review question. Different outcome definitions and measurement methods may lead to differences in study results and are a source of heterogeneity across studies and thus risk of bias. Occasionally a different definition of outcome is intentional to examine the usefulness of a model to predict alternative outcomes. For example, one may intentionally seek to validate for non-fatal events a model originally developed for predicting fatal events. A review of cancer prognostic models found that outcomes were poorly defined in 40% of the studies [60],[78]. It was often unclear whether mortality referred to cancer mortality or overall mortality from any cause, and in the definition of disease-free survival it was unclear which events were included.

In diagnostic modelling studies, establishing the presence or absence of the target disease is known as verification by a reference standard. Primary studies on the same target disease frequently use different reference standards which may have different accuracy for determining the true target disease status, potentially compromising the validity of study results; using a suboptimal reference standard may lead to misclassification of the target disease [79]–[81].

Some modelling studies use a combined outcome; for example, cardiovascular disease often comprises myocardial infarction, angina, coronary heart disease, stroke, and transient ischaemic stroke. A combined outcome is considered easily translatable to clinical practice or to achieve a higher effective sample size, but could lead to important predictors not being identified, as predictors may have opposite predictive effects in the component outcomes, causing their predictive contributions to cancel each other out. Reviewing and summarising predictors in models using combined outcomes is particularly challenging [82],[83]. In studies validating a prediction model for a combined outcome, the number and severity of individual component outcomes may differ markedly from the derivation study, potentially affecting the predictive accuracy of the model in the validation dataset [84]. When available, the systematic review should record the frequency of the individual components in the combined outcome to enable comparison across studies. If this information is not reported in the primary study and cannot be retrieved by contacting the study authors, then this should be reported in the systematic review.

In diagnostic studies the importance of assessing the reference test without knowledge of (i.e., blinded to) the results of the index tests is well established [35],[40],[54],[69],[79],[80],[85]. The same issue is also important in prognostic studies where the assessor of the outcome occurrence should be blinded to ascertainment of the predictor [47],[48]. In the absence of blinding, the predictive ability of the model may be overestimated because the predictors may be used in assessing the outcome. Blinded outcome assessment, in both diagnostic and prognostic studies, is most important when outcomes require subjective interpretation (e.g., results from imaging) that could be biased by knowledge of predictors. For so-called “hard” outcomes, such as overall mortality, blinded outcome assessment is less important. However, cause-specific mortality may include subjective interpretation so that knowledge of the predictors could bias outcome assignment. Several reviews have reported that many studies did not blind the outcome measurement for the candidate predictors [70],[81],[86],[87].

A special case of incorporating the predictor information in the outcome assessment is the use of so-called consensus or expert panel outcome assessments. This is often used in diagnostic studies for target diseases where the reference standard used in practice is known to include a subjective assessment of information [52],[54],[88],[89]. Here, a consensus panel typically uses all available information on the study participants, including the predictors (or index tests) under study, to determine whether the target disease is present. The results of the predictors are directly and deliberately incorporated in the assessment of the target condition, usually leading to optimistic predictive accuracy of the developed models. This specific form of non-blinded outcome assessment bias is commonly referred to as “incorporation bias” [52],[88],[89].

In the prognostic setting, retrieval of the follow-up period or a summary of the follow-up from the primary studies deserves special attention. Disappointingly, these key details are often poorly reported [72],[75]. A recent review found the number of participants with ten years follow-up was frequently not reported, even in studies validating prognostic models predicting a ten-year outcome [15].

#### Candidate predictors

Candidate predictors may range from simple patient demographics and clinical characteristics to advanced test results. We emphasise that *candidate* refers to the predictors chosen to be studied for their predictive performance, and not restricted to those included in the multivariable analysis [59]. The number of candidate predictors analysed in the primary studies is highly important. Together with the number of participants with the outcome (i.e., those with the event or the target disease) they contribute to the assessment of whether the model is likely to be overfitted. Overfitting occurs when idiosyncratic features of the development data attain spurious statistical significance and are retained in the final model: the model is too closely tailored to the data at hand [51],[58]. These models do not produce inaccurate predictions in the dataset from which they are developed, but they do when applied to other individuals. Predictions tend to be too extreme; low predicted risks will be too low and high predicted risks too high. Overfitting thus leads to models that are not transportable or generalizable.

Different definitions and measurement methods of candidate predictors are a potential source of heterogeneity and thus risk of bias, and the use of different measurement methods may affect the strength of predictors and influence whether the predictors ultimately are included in the prediction model [42],[61]. For example, type 2 diabetes mellitus, a known risk factor and therefore predictor for cardiovascular disease, can be defined by an oral glucose tolerance test, HbA1c measurement, fasting plasma glucose measurement, or even by self-report. These different predictors may have different predictive effects in the multivariable models. Also, models including predictors measured using routinely accessible equipment are likely more generalizable than predictors measured with less available techniques [61]. As with the outcome, the definition and measurement method of the predictors may sometimes be intentionally different when evaluating an existing model in a separate dataset. The review should highlight differences in definitions or measurement methods of any of the predictors, so readers can place the results in context.

Candidate predictors that can vary over time should be available and measured at the time of intended use of the prediction model, not at a later moment in time or after the outcome has occurred [47],[90].

As described for outcome assessment, measurement of predictors with knowledge of outcome information may inflate the predictive accuracy of the predictors and thus of the final prediction model. This concern particularly applies to predictors requiring subjective interpretation. In prospective studies, predictor assessment is inherently blinded, as it is completed prior to outcome occurrence. It may also be important to blind assessment of predictors to each other, particularly if a review seeks to address the predictive contribution of an additional subjective predictor beyond previously obtained predictors. For example, if the predictive ability of an MRI in addition to laboratory measurements is studied, the MRI should be interpreted blinded to the laboratory measurements to reduce possible bias [73],[91].

The methods used to handle predictors in the analysis can influence which predictors are selected for inclusion in the model and so affect model predictions. Continuous or categorical predictors are frequently dichotomised for the analysis [2],[42],[56],[60],[78] despite strong evidence and recommendations to the contrary [92]–[94]. Categorisation assumes a constant risk up to the cut-point and then a different risk beyond the cut-point, which is implausible and nonsensical. In addition, dichotomising discards information and commonly results in a loss of power [93]. Dichotomising predictors, particularly when choosing a so-called “optimal” cut point based on data from one study, often causes selection of spurious predictors and overfitting, reducing the reliability and applicability of model predictions in new patients [55],[93]–[96].

#### Sample size

One of the biggest concerns when developing a prediction model is the risk of overfitting. For dichotomous outcomes, overfitting typically arises when the number of individuals with the outcome (event or target disease) of interest is small relative to the number of variables. The number of variables includes all candidate predictors, transformations for continuous predictors, indicator variables for categorical predictors, and interactions examined. The number of events-per-variable (EPV) is commonly used to calculate the sample size, where attaining a sample size with an EPV of ten or more is frequently recommended to avoid overfitting [97]–[101]. For studies validating prediction models, sample size considerations are not well established, but a minimum of 100 events and 100 non-events have been suggested [102]. For continuous outcomes, 20 participants per predictor have been recommended [51].

A systematic review should therefore record both the number of individuals in the study and the number of individuals with the outcome or target disease. Numerous systematic reviews of prediction models have reported that the number of events per candidate predictor is often poorly reported and, when it is reported, that the EPV is often less than ten [2],[15],[56],[78].

#### Missing data

In all types of medical studies, including prediction modelling, some data is not available or not recorded. Differences between studies in the extent and type of missing data and the methods used to handle this missing data may greatly influence model development and predictive performance. Knowing the number of participants with any missing data across all included studies and whether these participants were included in model development or validation is important to understanding possible biases in prediction modelling studies. However, reporting on the frequency and type of missing data is often poor [2],[15],[56],[62],[78],[103]–[105] despite the adverse effects of missing data on development, validation, and updating of a prediction model [34],[103],[105]–[112]. These adverse effects are related to the amount of missing data [112] and the extent to which data are missing completely at random [108],[111]. Missing data are seldom missing completely at random; the missing data are often related to other observed participant data. Consequently, participants with completely observed data are different from those with missing data. A so-called complete-case analysis, which simply deletes participants with a missing value, thus leaves a non-random subset of the original study sample, yielding invalid predictive performance, both when developing and when validating a prediction model. Only if omitted participants are a completely random subset of the original study sample will the estimated predictor-outcome associations and predictive performance measures of the prediction model be unbiased [113]. Multiple imputation is generally acknowledged as the preferred method for handling missing data in prediction research. In this strategy, missing observations are substituted by plausible estimated values derived from analysis of the available data. However, when data are “missing not at random”, i.e., missing data is still partly due to unobserved data or characteristics of the participants, multiple imputation does not sufficiently solve the invalidity problem [107],[112],[113].

Detailed reporting in primary studies on whether missing data may reasonably be missing at random (by comparison of the participants with and without missing values) is invaluable for reviewers to judge the potential for bias. Numerous recommendations for reporting missing data in medical research have been proposed [103],[104],[114],[115]. It is therefore important during the systematic review to record from the primary studies whether the presence of missing data (how much and how handled) was mentioned.

### Model development

In appraising studies that include model development, first the type of model (e.g., logistic, survival, machine learning, other models) used should be assessed. It is important to summarise and understand key components that might lead to bias and variability between models. An important source of bias in model development is in the method of selecting the final predictors, especially in studies with a small sample size. We split the selection of predictors into two components, the selection of predictors for inclusion in the multivariable analysis and selection during multivariable modelling. The use of different predictor selection methods and criteria for predictor inclusion across studies may yield different models and different amounts of bias. These issues should thus be carefully documented during the review.

#### **Selection of predictors for inclusion in multivariable modelling**

In some model development studies, predictors are selected for inclusion in the multivariable modelling based on the association of each candidate predictor with the outcome. Although common, such screening or pre-selection based on univariable significance testing carries a great risk of so-called predictor selection bias [51],[56],[58],[116]. Predictor selection bias occurs when predictors selected for inclusion in multivariable modeling have a large but spurious association with the outcome. Including such predictors increases the likelihood of overfitting and thus over-optimistic predictions of a model's performance for other individuals. Furthermore, predictors that show no association with the outcome in univariable analysis because of small sample size may become associated with the outcome after adjustment for other predictors. The risk of predictor selection bias is greater in smaller datasets (when the EPV ratio is small), and when there are notably weak predictors.

Bias in predictor selection may also occur when continuous predictors are categorised. As discussed, it is recommended to keep continuous variables continuous and to check whether nonlinear transformations (e.g., using restricted cubic splines or fractional polynomials) are indicated [45],[51],[58],[93],[94],[96].

The systematic review should record how many candidate predictors were examined, any methods used to select predictors, and any methods used to transform predictors prior to inclusion in the multivariable analysis to assess risk of bias.

#### **Selection of predictors during multivariable modelling**

Just as the selection of predictors for inclusion in the multivariable modelling can contribute to optimistic and biased models due to overfitting, so can the selection of predictors during multivariable modelling. There is no consensus on the best method, but certain methods have been shown to be less useful and increase the risk of model overfitting, such as forward selection techniques [51],[55],[58],[117]. Two of the most commonly used methods are the “full model approach”, and “backward elimination”. The full model approach pre-specifies all predictors in the final model and no predictors are omitted, which avoids predictor selection bias [51],[58]. Whilst this approach sounds attractive, it requires substantive prior knowledge about the most promising candidate predictors [59], which is not always straightforward. Backward elimination starts with all candidate predictors in the model and runs a sequence of statistical tests to remove them from or keep them in the model based on a pre-specified criterion. Possible criteria for predictor inclusion include Akaike or Bayesian Information Criterion, the use of a nominal p-value (e.g., <0.05 based on the log likelihood ratio test in regression approaches), or using a change in the model's c-index (see below) [58]. The choice of a relatively small nominal significance level for predictor selection (e.g., p-value<0.05 or even <0.01) generates models with fewer predictors, but increases the chance of missing potentially important predictors, while larger levels (e.g., p<0.20 or p<0.25) increase the risk of selecting less important predictors. In both cases, overfitting may arise, particularly in small datasets [51],[55],[58],[59].

To address possible overfitting of a model, shrinkage techniques can be used to adjust the estimated weights of the predictors. The corresponding adjusted estimates of predictive performance are likely to be closer to the predictive accuracy that will be found when the developed model is applied to other individuals. Hence, studies that develop prediction models that are adjusted or shrunk are less prone to bias. The need for use of shrinkage methods increases with smaller datasets, although in datasets with a low number of EPV, even shrinkage methods cannot account for all bias [51],[58],[117],[118].

Given the strengths and weaknesses of various modelling and predictor selection strategies, the systematic review should record all information on the multivariable modelling, so readers can gain insight into how each model was developed.

### Model performance

Regardless of the statistical method used to develop or validate the model, various model performance measures such as calibration, discrimination, (re)classification, and overall measures of performance may be used [51],[58],[117]. Calibration and discrimination should always be recorded when reviewing clinical prediction models. Calibration refers to how well the predicted risks compare to the observed outcomes; preferably this is evaluated graphically by plotting observed against predicted event rates [51],[58],[119]. For time-to-event models using, e.g., Cox regression, calibration is usually evaluated at specific time points by comparing observed and predicted risks for groups of individuals [119]. Calibration plots are often supplemented by a formal statistical test, the Hosmer-Lemeshow test for logistic regression and its equivalent for Cox regression. However, such tests have frequently been criticised because of the limited statistical power to assess poor calibration and being oversensitive in large samples [58],[117],[120],[121]. Furthermore, the Hosmer-Lemeshow test gives no indication of the direction or magnitude of any miscalibration. Discrimination refers to how well the model differentiates between those with and without the outcome and is typically assessed using the *c*-statistic, which is the equivalent to the area-under-the-curve of a receiver operating characteristic curve. The *c*-statistic should not be used as the only performance measure, however, since it is influenced by the distribution of predictor values and is often insensitive to inclusion of an additional predictor in the model [59],[122]–[125].

Classification measures, notably sensitivity and specificity, may also be presented. However, the use of these measures requires a predefined probability threshold. The same model would show very different sensitivity and specificity depending on the chosen threshold. The reporting of performance based on thresholds chosen from the data itself can produce over-optimistic and biased performance [95].

Reclassification measures, such as net reclassification improvement or index (NRI), evaluate whether a single biomarker has any incremental value to a prediction model [124],[126]. Their use has been criticised as they rely on a priori–defined probability thresholds and do not account for difference in consequences of falsely reclassified individuals [122],[127],[128]. Furthermore, NRI is a measure of comparative performance and is therefore not directly useful as a measure of performance of a single model.

Recent systematic reviews have found the reporting of performance measures to be poor, with reliance on measures of discrimination [2],[15],[60]. Objective evaluation across multiple studies and models is difficult if other aspects of model performance are missing. Systematic reviews should ensure that if possible, at a minimum, aspects of discrimination and calibration are extracted. For a full appraisal of models across multiple studies, systematic reviews should also record whether the primary study actually evaluated both calibration and discrimination. The absence of either component makes a full appraisal of prediction models difficult.

### Model evaluation

When the predictive performance measures described above are evaluated or estimated in the same dataset used to develop the model, they are termed “apparent performance”. The apparent performance tends to be biased (i.e., overestimated relative to performance in other individuals). Regardless of which modelling technique (regression, neural network, or machine learning techniques) is used, this risk of bias is more pronounced when the development dataset is small, the number of candidate predictors is large relative to the number of outcomes, data-driven predictor selection techniques have been applied, and shrinkage techniques have not been used. The assessment of the performance of prediction models should not rely on the development dataset, but rather be evaluated on other data. In fact, evaluation in an independent dataset is all that matters; how the model was derived is of minor importance [49]. Quantifying model performance in other individuals is often referred to as model validation (Box 1) [1],[32],[49],[51],[56],[58],[59],[119],[129],[130]. Several strategies exist depending on the availability of data, but are broadly categorised as internal and external validation [1],[32],[49],[51],[56],[58],[59],[129].

Often the original dataset is randomly divided into a development sample and validation sample. However, this approach merely creates two similar but smaller datasets differing only by chance, and generally provides little additional information beyond the apparent performance, and for large datasets, the difference in performance in the development and validation dataset is even negligible, as expected [3],[32],[56],[58],[131]. Moreover, the method is statistically inefficient because not all available data are used to develop the prediction model, increasing the likelihood of overfitting, particularly in small datasets. Thus, for small datasets, the use of split-sample methods actually increases the risk of bias, whilst for large datasets there is no practical benefit [61]. If splitting the data is to be considered in large datasets, then a non-random split is preferable, for example splitting by time, centre, or geographic location [49],[51],[56],[58],[59],[129].

Internal validation using resampling (Box 1) is a method to estimate the amount of overfitting or optimism in the apparent predictive performance of the developed model, for which no other data than the original study sample is used. Internal validation by resampling quantifies bias in the apparent model performance. Rather than cross validation, bootstrapping resampling methods are generally regarded as the preferred internal validation method, as all the data is used for model development and for model evaluation. Regardless of the modelling technique, bootstrapping is particularly recommended in small datasets with many candidate predictors and when predictor selection techniques have been used [3],[49],[51],[55],[58],[59]. In addition to capturing optimism in model performance, bootstrapping provides a shrinkage factor to adjust the estimated regression coefficients and apparent model performance for such overfitting.

Preferably, the predictive performance of a model is quantified in data that were not part of the development study data, but external to it (Type 3, Box 1). External data can differ in time (temporal validation) or location (geographical validation) from the data used to derive the prediction model. Usually this second dataset is comparable to the first, for example, in patients' clinical and demographic characteristics, reflecting the target population of the model development study. Sometimes, however, it is of interest to examine whether a model can also have predictive ability in other scenarios. For example, a validation dataset may differ in the clinical setting of participants (e.g., primary care versus secondary care), in the age range of participants (children versus adults), in the clinical inclusion criteria, or even by using different predictor or outcome definitions and measurements [1],[31],[32],[56],[129],[132]. A crucial point is that a validation study should evaluate the exact published model (formula) derived from the initial data. Repeating the original modelling process in the validation data, refitting the model on new data, or fitting the linear predictor (in case a regression modelling technique was used) as a single term on the new data is not model validation, but rather model re-development [3],[32],[49],[51],[56],[58],[119]. If an existing model shows poor performance when evaluated in other individuals, researchers may adjust, update, or recalibrate the original model based on the validation data to increase performance. Such updating may range from adjusting the baseline risk (intercept or hazard) of the original model, to adjusting the predictor weights or regression coefficients, to adding new predictors or deleting existing predictors from the model [53],[58],[133]. Model updating, if done, usually follows an external validation of a previously published prediction model (Type 3, Box 1).

Systematic reviews should thus identify whether reported performance measures of the prediction models were obtained using only the development data (apparent performance), were corrected for optimism (e.g., using resampling techniques), used a random split-sample approach, or were based on performance in separate (external) datasets. If separate datasets have been used to develop and validate a prediction model, it is important to report any differences between the datasets. Updating or recalibrating a model based on external data should also be reported, if done. External validation studies provide the best insight into the performance of a model, indicating how useful it might be in other participants, centres, regions, or settings. However, many reviews have shown that external validation studies are generally uncommon [1],[12],[14],[15],[27],[29],[56],[78].

### Results

The results of the models in the review should match the systematic review question. If the aim is to review all existing prediction models in a particular clinical area, or for a particular outcome or groups of individuals (Box 2), results may include the components of the different models that have been developed, including the selected predictors, predictor weights, or regression coefficients (in case a regression approach was used) and their precision estimates, in addition to the performance of these models [12],[27],[29],[45]. If the aim is to review the reproducibility or predictive performance of the same model(s) across different study samples (external validation), as for example in [44],[134],[139], the predictive accuracy measures and their precision estimates are important to focus on, whilst issues surrounding the development of the models are less relevant to report.

As models are usually developed to estimate an individual's outcome probability, it is important to capture and record whether this can actually be done from the published model. The format used to present models in the original papers should be extracted. Options include the original model formula (e.g., the regression equation if a regression approach was used to develop the model) enabling direct probability estimation, rounded scoring rules, or predefined risk groups with corresponding predicted and observed outcome probabilities. Rounding or simplifying original predictor weights or regression coefficients is likely to cause a loss in predictive accuracy. Hence, if relevant, the systematic review should report the performance measures of the original and “rounded” models where this information is available in the published primary report [32].

Risk groups are frequently presented. For reasons described above, data driven methods to create risk groups, such as the “optimal” probability threshold method or at the median, are not recommended [135]. Therefore, it is important to note if and how risk groups were created. Recent reviews in oncology highlighted poor methods and poor reporting for creating risk groups [56],[60].

When a review includes both development and validation studies of the same model(s), or several external validations of the same model, reporting differences in frequency (binary) and distribution (continuous) of the predictors and outcomes across the study samples is recommended, as a different case-mix is known to result in different predicted risks that may influence model performance measures [49],[53],[129],[133],[136],[137].

### Interpretation and discussion

All tools for reporting of medical studies recommend discussing strengths, weaknesses, and future challenges of a study and its reported results [33]–[37], including the PRISMA statement for reporting of systematic reviews itself [38]. How a model was developed and validated and its reported performance give insight into whether the reviewed model is likely to be useful, and for whom. Conclusions about model performance and applicability should be based on the validation results of the model, the comparison with other studies and other prediction models, and study strengths and weaknesses, rather than predictor effects or corresponding p-values. Furthermore, one may like to overview the performance of all prediction models for a specific outcome or target population before making decisions on which model to apply in routine practice [138].

## Conclusion

In contrast to systematic reviews of therapeutic and diagnostic test accuracy studies, there is no formal checklist for guidance on defining a proper review question, let alone for data extraction or critical appraisal of primary studies on the development or validation of diagnostic or prognostic prediction models, despite the sharp increase of such studies in the past decade. We combined published risk-of-bias tools, existing critical appraisal checklists for systematic reviews of randomised therapeutic trials and diagnostic test accuracy research, methodological recommendations for conduct and reporting of prediction model research, and data extraction sheets used in published reviews of prediction modelling studies to provide the CHARMS checklist. The checklist is intended to help frame the review question, design the review, and extract the relevant items from the reports of the primary prediction modelling studies and to guide assessment of the risk of bias and the applicability of the reviewed prediction models. We recognise that this checklist will require further evaluation and use to adjust and improve CHARMS.

## Supporting Information

Text S1 A one-page checklist of relevant items to extract from individual studies in a systematic review of prediction models.
(DOCX)
